# A Review of Functional and Structural Neurobiology of the Action Observation Network in Autism Spectrum Disorder and Developmental Coordination Disorder

**DOI:** 10.3390/brainsci9040075

**Published:** 2019-03-28

**Authors:** Emily Kilroy, Sharon A. Cermak, Lisa Aziz-Zadeh

**Affiliations:** 1 Mrs. T.H. Chan Division of Occupational Science and Occupational Therapy, University Southern California, Los Angeles, CA 90089, USA; cermak@usc.edu (S.A.C.); lazizzad@usc.edu (L.A.-Z.); 2 Brain and Creativity Institute, University Southern California, Los Angeles, CA 90089, USA

**Keywords:** Autism spectrum disorder (ASD), developmental coordination disorder (DCD), action observation network (AON), functional magnetic resonance imaging (fMRI), resting state, diffusion weighted imaging (DWI)

## Abstract

Recent research has reported motor impairment similarities between children with developmental coordination disorder (DCD) and a subgroup of individuals with autism spectrum disorder (ASD). However, there is a debate as to whether DCD is a co-occurring diagnosis in individuals with ASD and motor impairments (ASDd), or if motor impairments in ASD are distinct from DCD. However, the etiology of motor impairments is not well understood in either disorder. Clarifying comorbidities in ASD is important to determine different etiopathological phenotyping clusters in ASD and to understand the variety of genetic and environmental factors that contribute to the disorder. Furthermore, this distinction has important therapeutic relevance. Here we explore the current neuroimaging findings in ASD and DCD and discusses possible neural mechanisms that underlie similarities and differences between the disorders.

## 1. Introduction

Autism spectrum disorder (ASD) is defined clinically by the presence of social communication and social interaction impairments, along with restricted and repetitive behaviors including motor movements, insistence on sameness, and/or atypical sensory processing [1]. An ASD diagnosis may be complicated by one or more co-occurring conditions, including anxiety, attention deficit hyperactivity disorder (ADHD), epilepsy, Fragile X syndrome, neuroinflammation and immune disorders, sensory processing disorders, obsessive-compulsive disorder, and/or developmental coordination disorder (DCD), all of which make ASD a highly heterogeneous condition that is difficult to study. Overlapping symptoms from comorbid diagnoses can mask and interact with ASD-specific symptoms. Due to the heterogeneity of ASD research participants, historically it has been challenging to identify discrete ASD behaviors and neurological patterns. In particular, motor impairments constitute a comorbidity that affect a large percentage (80–90%) of children with ASD [2,3,4]. Indeed, imitation and motor deficits have been associated with ASD since Kanner’s initial description of the disorder [5]. Nevertheless, they are not part of the diagnostic criteria for ASD nor are they commonly measured and considered a confounding factor in neuroimaging studies on ASD.

Given the high comorbidity rate of motor deficits with ASD, it is difficult to distinguish the impact of motor impairments on the social deficits that are central to ASD. For this reason, here we review neuroimaging literature on a second developmental disorder primarily characterized by motor difficulties, DCD (sometimes referred to as dyspraxia), and relate it to similar literature on individuals with ASD. Individuals with DCD have difficulty in motor learning and poor motor performance [1] as well as challenges with motor imitation [6]. Indeed, motor and social skills are influenced by the function, structural integrity, and connectivity of motor brain regions and networks in humans. However, how motor ability and motor perception interact with social ability and social perception in ASD is unclear. Because individuals with DCD have primary impairments in motor skills but not social skills [1,7,8,9,10,11,12], they can act as a comparison population to individuals with ASD with and without a co-morbid DCD diagnosis to help disentangle potential interactions between motor performance and social processing deficits in ASD, as well as investigate possible distinct ASD motor impairments.

Several reviews on motor networks in ASD and a few on DCD have been published, however, to our knowledge, no neuroimaging reviews to date have compared these populations side-by-side. It has been argued that some motor regions of the brain, such as the action observation network (AON), may be compromised in ASD in a manner that can lead to both imitation and social deficits in this population [13,14,15,16]. The same network has been implicated in DCD motor impairments [17]. AON dysfunction has been tied to both function and structural integrity in these populations. Thus, here we will focus the review on current AON literature across multiple levels of neurobiology as it relates both the ASD and DCD populations in order to better understand how the AON varies in these groups. After briefly describing the AON, DCD, and behavioral motor and imitation challenges in the two clinical groups, we will look at evoked and resting state functional magnetic resonance imaging (fMRI) data followed by white matter microstructure findings. We will conclude by discussing results and future directions for research. 

### 1.1. The Action Observation Network (AON)

**Figure 1:**
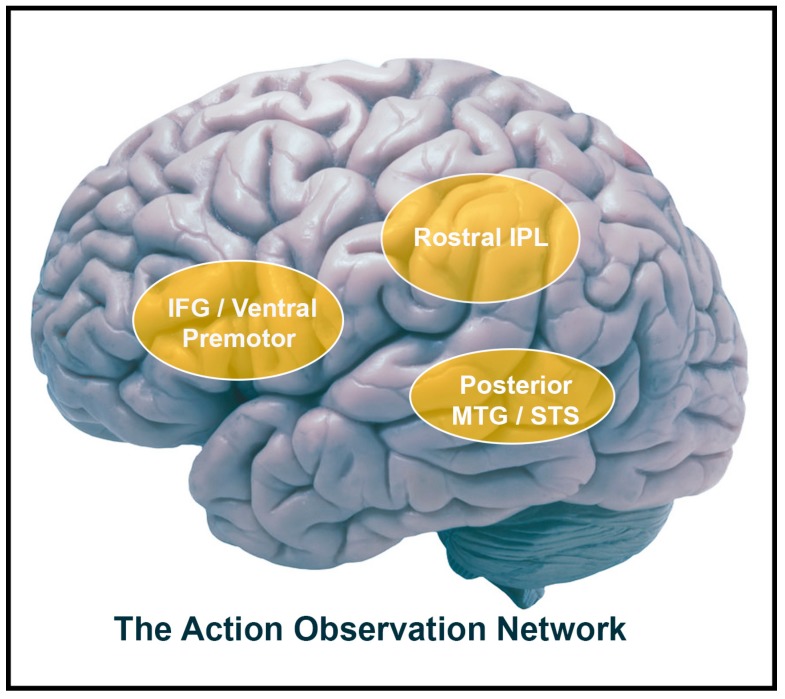
Action observation network components. Lateral view of brain with frontal (ventral premotor and IFG) and parietal (rostral IPL) labels of the mirror neuron system in addition to the superior temporal sulcus. IFG = inferior frontal gyrus; IPL = inferior parietal lobule; MTG = middle temporal gyrus; STS = superior temporal sulcus.

By observing other people’s actions, we often can obtain important social information regarding their goals and intentions. Observing others’ actions elicits activation in multiple sensorimotor brain regions that collectively comprise a network called the AON [18,19,20]. The AON’s core regions include the inferior frontal gyrus (IFG), premotor cortex, and inferior parietal lobule (IPL), as well as temporal areas such as the posterior middle temporal gyrus (pMTG) and the superior temporal gyrus (pSTS). The premotor cortex, the inferior frontal gyrus (IFG), and regions of the IPL have been shown to be active during both the execution and observation of a given action [18,21,22,23]. These regions are thought to be the human homologues to the brain areas in monkeys in which “mirror neurons” originally were identified [24]. Accordingly, these human brain regions are thought to comprise the putative human mirror neuron system (MNS [24]; Figure 1). Typically, the MNS is thought to be a distinct part of the AON because it is active both during the production and the observation of actions [25,26] while the AON can constitutes brain regions that are involved in action observation, but not necessarily in execution. Thus, in this review, the AON is defined as a broader network of regions involved in action observation, specifically: the IFG, premotor cortex, IPL, and temporal regions.

It has been hypothesized that the AON contributes to the understanding of others’ actions by mapping those actions onto one’s own motor system [24]. In a sense, the AON implicitly simulates a corresponding motor representation, which could help process the goals and intentions of other people’s actions [27,28]. Because the AON may be important for goal and intention understanding in others, it has been related to social cognition, such as components of empathic processing [27,29,30], which has led some to implicate the network’s role in social disorders such as ASD [14]. Indeed, the AON’s function and connectivity at rest and its underlying white-matter integrity have been linked to behavioral measures of social and motor skills [31,32]. Many researchers posit that the AON is crucial to social cognition [14,33,34,35], while others have argued its primary role is in motor perception but not action understanding [36,37,38].

Brain imaging studies on the AON in ASD have yielded contradictory findings. Some report attenuated AON activity in ASD [14,28,39,40] while others have reported intact functioning [41,42,43,44]. Furthermore, since social and motor deficits are often linked in ASD [4,45,46], it is difficult to understand how these deficits may interact within distributed networks. One way to try to answer this question is to compare individuals with ASD to individuals with DCD, as both disorders often share similar motor impairments, while social impairments are more singularly endemic to ASD. Through this type of comparative analysis, we may be able to tease apart ASD’s social impairments from its motor symptoms. 

### 1.2. Developmental Coordination Disorder (DCD) and Imitation Impairments 

Developmental coordination disorder (DCD) is a neurodevelopmental disorder that affects approximately 6% of the school-age population (APA, 2013). Individuals with the disorder have poor motor skills that interfere with the ability to perform everyday activities. To receive a DCD diagnosis under the DSM-5, these deficits “cannot be explained by sensorimotor impairments sufficient to preclude skilled movement” nor by intellectual disability [1]. Although cognitive and psychosocial impairments are not primary symptoms in a DCD diagnosis, individuals with DCD may demonstrate challenges in social functioning, self-esteem, and psychological adjustments which are presumed to result from DCD symptomatology (e.g., not being selected for sports teams, and teasing due to motor disability [8,9,11,47]. A recent behavioral study places DCD socialization skills at an “intermediate” level between typically developing children and those with ASD [48]. These secondary social symptoms can put individuals with DCD at higher risk for depression and anxiety [10]. The disorder can also co-occur with ADHD, specific language impairment (SLI), and ASD [49,50].

Among DCD’s myriad of motoric challenges (dressing, handwriting, sports, etc.), behavioral data also indicate the presence of imitation impairments [51,52]. Individuals with DCD make more errors and have slower response times when imitating learned or meaningful skills [6,53,54] as well as when imitating “non-meaningful” simple and complex gestures [52,55]. Such deficits may be minimized, however, for very common and learned gestures such as waving goodbye. Investigating the imitation of common representational gestures (e.g., waving goodbye, brushing teeth), one study found no differences between children with DCD and typically developing (TD) peers [10], demonstrating that individuals with DCD are capable of imitating over-learned motor actions. Moreover, while there is substantial evidence that motor imagery (MI) is impaired in individuals with DCD there is preliminary evidence to suggest the efficacy of MI, or the mentalization of actions, for treating DCD [56,57] providing further evidence that individuals with DCD may have the ability to overcome some motor impairments with training. Conversely, there is evidence to support that imitation deficits extend to novel and sequential gestures [55,58,59,60,61]. Given that individuals with DCD can effectively learn common gestures, it may be more appropriate to utilize novel gestures when assessing imitation abilities in DCD. Thus, caution is advised when interpreting results from imitation studies that rely exclusively upon over-learned representational gestures in the study design. Unfortunately, the use of standardized non-representational assessments is limited in the literature, although the Postural Praxis subtest within the Sensory Integration and Praxis Tests (SIPT [58]) does incorporate novel hand and body gestures, and the Florida Apraxia Screening Test Revised (FAST-R [62]) includes novel gestures along with more common representational actions, and these assessments have been used with some consistency [52,63,64]. Future experimental designs would benefit from incorporating imitation of novel gestures to more accurately test and reflect imitation skills, rather than relying on representational gestures alone.

Heterogeneity of deficits in DCD has been well established [65] and should also be considered when interpreting results. It is thus possible that different subtypes of DCD vary in their levels of imitation impairment. Many efforts have been made to characterize DCD subtypes, although few have focused on imitation as a predictive variable. Different approaches to subtype DCD include: cluster analysis of different motor skills [66,67,68,69,70], cognitive motor abilities [71], and presence of minor neurological dysfunctions (MNDs [72]). One subtype of DCD has been tied to imitation, Vaivre-Douret and colleagues [72] found imitation to be a key predictor of an ideomotor DCD (IM-DCD) subtype. They used a set of neuropsychological testing (Neuropsychomotor test [NP-MOT]) and multivariate statistical techniques to identify patterns of DCD subtypes. Among the different variables, imitation (non-meaningful hand and finger imitation of gestures), digital perception (localization of digital tactile stimuli), and digital praxis were found to be primary variables in identifying those with DCD with ideomotor deficits compared to those with visuo-spatial/ constructional (VSC) impairments [73]. It is unknown how the neurological underpinnings of this disorder vary across subtypes. To date, only a few neuroimaging studies have investigated imitation and fine motor skills in individuals with DCD, none of which compared subgroups of the disorder. Overall, further research is needed to understand how variation in motor deficits corresponds to neural mechanisms in individuals with DCD and how they respond to therapy.

### 1.3. Motor and Imitation Impairments in ASD

Motor abnormalities in ASD vary in severity. Impairments described by Kanner included clumsiness in gait and motor performances [74,75,76]. Since then, research has produced a growing body of evidence demonstrating that motor abnormalities including gross and fine motor functions, the inability to execute a sequence of actions (apraxia, difficulties in imitation), motor imagery and deficiencies in motor learning are prevalent in individuals with ASD [2,10,77,78,79,80,81,82]. Furthermore, sensory-motor impairments indicative of MNDs have also been reported in children with ASD. It has been observed that children with ASD have a wider range of impairments in fine manipulation, sensory deficits, and choreiform dyskinesia compared to typical peers [81]. These findings suggest sensory-motor in ASD may be linked to prenatal, natal, and natal risk factors [81]. However, despite the increasingly growing body of motor research conducted in ASD, no particular motor symptomatology is uniquely identified as ASD [83]. Nonetheless, several researchers have put forth predictions for the utility and impact of motor assessments for diagnostics and/or outcome measurements in ASD [84,85,86,87,88]. Motor assessments utilized as a diagnostic tool would be ideal since motor dysfunctions are persistent across development [89,90,91,92] and consist of quantifiable metrics that can more easily be detected and measured than social impairments. While there are several motor impairment measures available, reliability and validity has not been well established in ASD populations (For a review see Wilson et al., [93]).

Imitation deficits have also been implicated in ASD [40,64,84,94,95,96] though not all researchers agree with this. Some have investigated imitation in individuals with ASD and have found imitation to be either typical [97,98,99], or even enhanced [100,101]. Indeed, the ability to imitate can remain intact, depending on context and type of imitation [102,103,104]. Overall, there is evidence that individuals with ASD fail to mimic meaningless actions [105] or gestures [106] but perform typically on a range of other imitation tasks, such as common gestures, especially if they are goal-directed [105,107,108]. Hamilton [103] posits that goal-directed imitation is intact in ASD, whereas spontaneous mimicry of low-level kinematic features of action such as hand gestures or facial expression is impaired (mechanism regarding this theory are discussed in the *Neural Theories of Aberrant Findings in ASD* section below). These studies illuminate the complexity of social and motor deficits in ASD and demonstrate the need for special considerations of the type and level of imitation and motor skills involved when conducting and interpreting imitation research. 

### 1.4. Imitation Impairments in ASD and DCD

To date only one study has investigated how DCD subtype profiles correspond to individuals with ASD [109]. Paquet and colleagues (2019) observed that motor similarities between ASD and DCD were not associated with the predictive diagnostic markers that characterized subtypes of DCD (IM-DCD, VSC-DCD, or mixed-DCD (MX: both IM and VSC characteristics)). Instead, motor deficit variables differed among the groups but children with ASD performed overall better than those with DCD. However, when comparing imitative skills, children with ASD had greater difficulty with gestures using hands and fingers than those with VSC-DCD (significant difference) and the MX-DCD (non-significant difference) group. Moreover, they observed that ASD performed better with familiar and meaningful actions associated with an object than with imitative, non-meaningful actions. This finding is congruent with findings from a review on imitation in ASD conducted by Williams et al. [96]. However, in an earlier study [110], the opposite pattern (ASD worse than DCD) was found when ASD and DCD participants were compared on non-representational gestures as measured by a modified gesture task [111] and the MABC [112]. Over all, both ASD and DCD groups reported greater difficulty in making representational gestures than in non-representational gestures but children with ASD were less efficient than children with DCD at performing both types. On the MABC, they found slight disadvantages on the manual dexterity and the balance task but significantly worse on the catching and aiming subscale. It is possible that heterogeneity in samples lead to these two studies disparate findings.

The question of whether individuals with ASD have a discrete or comorbid ASD and DCD diagnosis has been an ongoing discussion, yet the answer is no clearer. Several hypotheses have been put forth suggesting neural mechanisms dysfunction surrounding imitation and gesture impairments in ASD such as the MNS/AON [40,96], hemispheric laterality [109,113], parietal-temporal and executive function [105,114], and visual integration [87,115]. 

The limited neuroscience research investigating motor impairments in ASD and DCD as well as inconsistent use of assessments makes understanding the specifications of motor impairments in these two groups difficult. The complexity of neural functioning and the heterogeneity in both groups only compounds the issue. Basic mechanisms well understood in individuals without neurological disorders is a promising place to start expanding our understanding of the disorders. Because components of the AON have been found to be essential for imitation [116,117], it is possible that the imitation deficits seen in DCD are tied to AON function as they have been proposed to be in ASD [14,64]. Understanding the AON in both groups may provide important information in determining discrete and overlapping neural signatures of motor impairment. A small but quickly growing body of literature has begun to directly and indirectly identify atypical AON functioning within the DCD population [17,118,119,120,121]. In later sections, we review the findings from fMRI studies focusing on the AON in ASD and DCD populations followed by examination of resting-state and diffusion literature in both populations to understand the network in these populations across different levels of neurobiology.

## 2. Task-Based AON Research 

In the last few decades, a considerable body of literature has examined the neural substrates that correlate with observation, execution, and imitation skills in typical adults (for a review see Caspers et al. [18]) and in ASD populations [40,122]. To date, no experimental research published on the neural mechanisms that underlie action processing in clinical populations have contrasted the ASD and DCD populations. Instead, studies examined each group separately or in comparison to either typical controls or other developmental disorders such as ADHD [52,123]. Below we review the task-based fMRI literature on studies related to the AON as observed in both the ASD and DCD populations and discuss how these findings inform AON functioning in both groups.

### 2.1. AON Activity in ASD

Dysfunction of the AON/MNS in relation to ASD has been coined the “broken mirror hypothesis” of ASD [124]. This hypothesis implies that ASD symptoms such as imitation and social cognition are related to the dysfunction of these mirroring areas which are crucial to imitation and social cognition [34,35,40]. Imitation is thought to be foundational to social development and important for understanding others and for communication [125]. Williams et al. (2001) was one of the first groups to hypothesize that deficits in the AON/MNS may contribute to imitation and social impairments in ASD. Indeed, there is a link between imitation and social skills such as empathy [14,29,126]. Moreover the close proximity and white matter tracts between emotional brain regions such as the insula or anterior cingulate and the AON (frontal and parietal areas) has led some to propose that these two networks (the AON and emotion-related networks) work together to support empathy and social cognition through implicit imitation and/or simulation [125,127]. These hypotheses are supported by a seminal study which demonstrate reduced activation in components of the AON (e.g., IFG) in individuals with ASD while observing and imitating static images of emotional expressions compared to TD children and adolescents [14]. The authors reported that reduced activity in the IFG during observation and imitation tasks was related to ASD symptoms as measured by ASD clinical assessments (e.g., Autism Diagnostic Interview Revised; ADI-R). As the signal decreased in the IFG pars opercularis (IFGpo), the social severity scores increased indicating that MNS dysfunction relates to social symptoms in ASD.

Other groups have replicated this effect in children and adults with ASD using different emotional stimuli. Grèzes et al. [128] reported reduced AON activity in adults while viewing neutral or fearful whole-body human actions, however, the authors attributed this reduction to decreased activity in emotion-related areas of the brain (i.e., amygdala) driving attenuation of network connectivity and activity in other brain areas such as the IFG [128]. In contrast, others report typical AON responses in ASD [129]. For example, in a study by Schulte-Rüther et al. [129], TD and ASD participants observed and responded to happy and sad facial expressions and were asked to either “decide how this person feels” or “decide how you feel when you look at the face”. Thus, the task in this study differed from the Dapretto et al. [14] study described previously, in that it did not involve passive observation or imitation. However, in this study, both groups activated left hemisphere IFG when instructed to think about their own emotions, which suggests that, in some instances, the IFG in ASD functions similarly to TDs. However, group differences were found in regions elicited to understand the thoughts and feelings of others known as the theory of mind network (ToM: medial prefrontal cortex (mPFC) and temporal parietal junction (TPJ)). Furthermore, several fMRI studies have reported no attenuation of activity in the mirroring regions in ASD compared to TD peers when asked to observe and imitate non-emotional actions such as finger tapping [43,44,52,100,130,131,132]. These findings go against the theory of global disruption of the AON and indicate that AON function may not be directly linked to poor imitation or social processing in ASD. However, these findings may be confounded by variability in imitation skills in the ASD samples. Since there is a large amount of heterogeneity in the ASD population with regards to motor ability, correlating results with motor ability assessments is an important consideration. Otherwise, it is possible that the differences in results observed between studies could be due to differences in the motor ability of the ASD samples in each study. It may be that the atypical IFG function is associated only with individuals who have motor impairments. Accordingly, studies that include individuals with motor deficits may find differences while those that incorporate participants who have more typical motor skills do not find such differences. 

Indeed, there is some evidence that atypical motor circuitry in ASD is related to poor motor skills. Mostofsky et al. [133] observed activation and connectivity differences in motor circuitry during a bilateral finger tapping task between ASD and TD children. In a follow-up study, functional seeds from the finger tapping task in the control group were used to investigate connectivity in TD and ASD participants [134]. Children with ASD showed rightward lateralization which was associated with poorer performance on motor skills measured by the Physical and Neurological Exam for Subtle Signs (PANESS) [135] which measures motor control among other clinical subscales. Interestingly, no association was found between motor connectivity and social and communicative sub-scores on clinical ASD assessments (e.g., Autism Diagnostic Observation Schedule; ADOS) [136] or ADI-R; Floris et al., [134]). These findings indicate that some motor deficits in ASD may not relate to social deficits. Furthermore, the type of stimuli used may contribute to conflicting study results—more socially relevant stimuli (facial expressions) may show greater AON differences between ASD and TD groups than less socially relevant stimuli (i.e., finger tapping). Finally, conflicting results could be due to differences in age of participants [33,137]. Previous work has observed age-related increases in inferior frontal gyrus activation in individuals with ASD [33]. Overall, the neural correlates that underscore social and motor skills in ASD remain unclear. Most studies have failed to examine both social and motor skills or how they relate to the neural underpinnings of AON function, which makes it difficult to test if variance in these skills accounts for the aberrant neuroimaging findings found in ASD.

#### Neural Theories of Aberrant Findings in ASD

Looking primarily at fMRI studies, there is no clear conclusion regarding the link between AON function and ASD symptomatology. Here we briefly list proposed explanations for aberrant AON findings. The first explanation is the multiple route theory put forth by Hamilton and colleagues [138,139] which proposes that multiple routes (direct and indirect) underlie imitation in humans [138]. The direct route (IFG to middle temporal gyrus [(MTG]) is thought to be responsible for spontaneous imitation and an indirect route (IFG via IPL) for emulation of goal directed actions. Based on behavioral imitation studies, Hamilton [139] suggests that the direct (mimicry) route is disrupted in ASD whereas the indirect (goal processing) route is intact. More recently, Hamilton and colleagues proposed that disruptions in social processing regions (such as the mPFC) known to be active during ToM might inhibit AON functioning in ASD through top-down control. The social top-down response modulation (STORM) model by Wang and Hamilton [140] hypothesizes a disruption in visual-motor mapping and the top-down modulation system is associated with mimicry deficits in ASD. Wang and Hamilton [140] posit that imitation ability is intact in ASD but being able to modulate imitation implicitly based on ToM processing in social situations may be impaired. This theory fits with findings of atypical activation of both ToM and AON regions [129]. In a follow up study, Hamilton and colleagues [141] measured imitation after direct and indirect eye gaze and found that mimicry was intact in ASD participants and was not modulated by gaze type. Still others have suggested a mixed partially “broken mirrors” and STORM theory (see Jiménez, Ortiz-Tudela, Méndez, and Lorda [142]) indicating deficits in both the mirror neuron system and social top-down mechanisms.

Several other studies also have reported deficits in other networks that are linked with the AON and that could result in atypical AON activity [44,122,143]. For example, in an activation likelihood estimation (ALE) meta-analysis of observation and imitation fMRI studies in ASD, decreased activation in frontal regions such as dorsolateral prefrontal cortex (DL PFC) were identified in ASD compared to a TD cohort [122]. The DLPFC has recently been implicated in rule-based visuomotor associations when observing actions [144]. During a simple social interaction, such as producing a rule-based motor response to the movements of another individual, the DLPFC was found to be crucial for modulating automatic imitative responses. The DLPFC is also important for attention and executive function [145,146], therefore those with attention deficits may have different AON responses.

The reward system also is known to be linked with AON functioning [147,148,149] and disrupted in ASD [150]. In neurotypical participants, reward sensitivity has been reported to mediate the association between IFG activity and task performance on a go/no-go task. Connectivity between the IFG and the ventral striatum (part of the reward circuit) has been reported to be related to ASD traits when viewing faces [151]. The authors posit that the reduced spontaneous mimicry of social stimuli seen in ASD may be related to the reward system’s failure to modulate the AON rather than a circumscribed deficit in the AON itself [151]. Evidence supporting the reward system-AON relationship has led some to suggest the perceived value of the action may modulate AON function [152]. Therefore, individuals with ASD who have higher social skills may find a social reward stimulus more valuable, and therefore have higher levels of AON activation. The notion of a value-based AON link is supported by findings from an electroencephalogram (EEG) study examining mu suppression, a putative marker of ‘mirror neuron’ functioning, while children with ASD observed the actions of familiar and unfamiliar people. Results revealed that children with ASD exhibited relatively typical AON activation while observing the actions of familiar individuals (more valued) but decreased AON activity when observing the same actions made by unfamiliar (less valued) individuals compared to TD peers. Future studies comparing the subjective value of social and nonsocial stimuli would be useful to determine if the AON is sensitive to a broader range of actions.

Evidence from other fMRI studies suggests that a possible disruption in ASD brain function may lie in the visual-motor system pathways. Lestou, Pollick, and Kourtzi [153] suggest that different regions of the broader AON (visual areas, and the STS) are involved with different aspects of action observation. Lestou and colleagues propose a bi-directional pathway between the STS and core MNS such that representation of action in the STS influences motor planning in the parietal and premotor regions of the MNS as well as motor plans in the ventral premotor cortex which change to predict actions in the STS. Indeed, disruption in visuomotor pathways has been reported in ASD. Poulin-Lord et al. [154] showed that in a visuomotor imitation task, ASD and TD participants demonstrated increased spatial variability of peak activation in the occipital cortex and MTG, as well as increased activation in the precuneus and superior and mPFC compared to peers. Interestingly in a recent behavioral study, coordination difficulties in children with ASD were linked to visual processing impairments while coordination impairments in DCD were linked to spatial processing [155] as measured by the Sensory Profile Checklist Revised second edition [156]. Despite these discrete sensory profiles, to date no neuroimaging study has compared the neural correlates of visual pathways between ASD and DCD. 

To conclude, there is a debate as to whether impairments in imitation seen in ASD may not be the result of a single network such as the AON (Broken Mirror Hypothesis [35]) or whether different networks and mechanisms such as executive networks, reward system visual and other sensory motor network disruptions may influence the AON. Furthermore, as we noted in the previous section, heterogeneity within the ASD population calls for correlating and controlling neural patterns with measures on both social and motor ability. Deep phenotyping of populations as well as effective connectivity analysis used to infer directionality of information transfer may help to better understand the relationship between these networks in the future.

### 2.2. AON Activity in DCD

There are fewer imaging studies designed to investigate the AON system in DCD than in ASD. To our knowledge, only three studies directly measured mirroring properties of imitation in DCD [52,120,157] while others measure gestures and imitation in general and provide indirect insight into the AON system. Research conducted using fMRI and EEG techniques to explore AON activation while participants completed hand and fine motor tasks (e.g., trail-tracing task adapted from the original MABC) report decreased activation in AON regions [17] as well as other motor-related regions (cerebellar, parietal, and prefrontal networks) in DCD relative to same-age peers [17,118,158,159]. Findings from a recent ALE meta-analysis of neural activation during manual dexterity tasks in DCD supports AON accounts of DCD by highlighting reduced activation in the supramarginal gyrus, and IPL [121]. Moreover, a recent single-pulse transcranial magnetic stimulation (TMS) study investigating mental imagery in young adults with DCD found that those with DCD performed on a mental imagery task compared to healthy controls and there was evidence that they had less activity in the primary motor cortex during mental imagery [160]. Overall findings suggest there are differences in motor brain function of children with DCD compared to TD children (see Wilson et al. [88] for review) with some exceptions (see Reynolds, Billington, et al. [161]). Limitations to synthesizing results across studies include the use of different fMRI task protocols and different assessments to measure motor function. One very common measure of motor performance included in fMRI studies is the Movement Assessment Battery for Children, Second Edition (MABC-2 [162]; Table 1). However, the MABC-2 does not assesses non-representational gestures. Instead, it provides measurements of motor skill and coordination. As mentioned above, the Sensory Integration and Praxis Tests [58] include one subtest of non-representational gestures (Postural Praxis), and the FAST-R and the Florida Apraxia battery (FAB [62]) both contain non-representational gestures in their respective imitation subsections while also including tasks that more closely resemble those typically designed for action observation fMRI studies, however these were not commonly used in previous studies leaving a critical gap in imitative assessment of these populations [120,161,163,164].

The three studies that have directly investigated the AON in DCD have one notable limitation, they all have small samples [52,120,157]. In a study by Licari and colleagues [157], 13 children with DCD and 13 age-matched TD peers were scanned while performing finger sequencing and hand clenching tasks. During the finger sequence task, the DCD group activated the left superior frontal gyrus and IFG less than the TD group and activated the right postcentral gyrus more than the TD group. No group differences were found in the hand clenching condition. While the decreased activation of the IFG is consistent with the broken AON hypothesis, increased activation of the postcentral gyrus contrasts with other motor related DCD findings. In a study not directly investigating the AON, Zwicker et al. [17] found decreased activation in the left postcentral gyrus (in addition to reduced IFG activation) in children with DCD during a trail-tracing task. The authors also reported increased activations in a number of other attention-related brain regions activated during the task (e.g., posterior cingulate gyrus, dorsolateral prefrontal cortex) and hypothesized that participants in their DCD sample required more attentional effort to perform the same task than did the typical sample. This postulation is supported by a study which reported that children with ADHD (a common comorbid disorder in DCD) performing a finger tapping task were observed to have decreased activation in primary motor areas and superior parietal cortex [64]. Together these findings suggest that other attention networks may influence motor and imitation processing in DCD.

Using a different finger sequencing task to explore observation and imitation in DCD, Reynolds, Licari, et al. [120] similarly reported decreased activity in the left IFG in 14 children with DCD compared to 12 age-matched TD controls. Reynolds and colleagues demonstrated that motor planning (measured by the Postural Praxis subtest of the SIPT; [58]) was positively correlated with IFG activation, which indicates that motor planning and imitation skills are related to AON dysfunction in DCD. However, no data on social skills was collected or reported to determine if the activity was collinear with social impairments. In a follow-up study Reynolds, Billington, et al. [161] compared 10 children with DCD to nine TD controls during observation, motor imagery, action execution and action imitation and found no differences in AON activation during a finger tapping test. The results of this study do not provide support for the AON dysfunction theory as a possible causal mechanism for DCD, although the sample size was extremely small. Ideally, a sample size at least double to the one used by Reynolds, Billington, et al. [161] is preferred [165]. Smaller sample sizes, especially in fMRI studies, tend to be statistically underpowered and have inflated number of reported foci [165]. Regardless, the authors suggest that other networks contribute to the imitation deficits seen at the behavioral level such as attentional networks and motor planning processes.

Notably, there are several methodological limitations to these DCD fMRI studies. As previously mentioned, most studies have very small sample sizes (Table 1). Furthermore, not all correct for multiple comparisons which now is considered standard practice in MRI analysis [166]. In addition, not all of these studies control for confounding effects of demographic or clinical variables e.g. sex, age, IQ; see Wilson et al., [56] for a detailed list. In short, while these initial papers provide valuable data on DCD neurobiology, these methodological limitations make validity and consensus about the neural basis of DCD difficult to ascertain.

### 2.3. AON Activity in ASD and DCD

Conclusions regarding AON task-based function in ASD and DCD are difficult to reach given the aberrant findings in both populations as well as the limited number of studies and small sample sizes, especially in DCD research. Overall, there is evidence both for and against AON dysfunction in both groups, especially in the IFG. The strongest evidence of AON dysfunction in ASD comes from studies that utilize more socially relevant stimuli [14,128]. These social-related imitation findings may be the result of impaired social deficits and/or motor deficits [140] and are difficult to tease apart. 

With regard to DCD, a few studies observed reduced AON activity [120,159,167]. Since motor skill assessments were not collected or analyzed in most task-based ASD studies nor were social skill assessments related to neural activation in the DCD studies, it is difficult to know for sure whether the attenuated AON function observed in both groups has a common source or is expressed differently in each group. To our knowledge, no research in ASD or DCD has compared imitation of both social (e.g., emotional expressions) and non-social (e.g., hand actions) actions. A potential future study that could clarify the specifics of AON dysfunction in these groups would compare both populations while they observed, executed, and imitated both social and motor actions. Contrasting more socially related actions to more motoric actions would illuminate the co-occurring effects of social and motor deficits in the AON and any possible additive effects of both diagnoses. Further, deep characterization of participants would also allow for a better understanding of activity patterns across motor and social networks in subtypes of participants. Finally, activity patterns elucidated from the study could be used to correlate with individual differences in behavioral measures such as meaningful and non-meaningful imitation skills. Ultimately, this research would help to understand the neural bases of variation in ASD and provide data to inform the development of optimal behavioral interventions tailored to the abilities and challenges of each individual with ASD as well as with individuals with DCD. 

#### AON Connectivity in ASD and DCD During Tasks and at Rest

Although looking at activity in specific regions of a neural network, such as the AON, can provide substantial information about potential deficits in ASD or DCD, human cognitive function is generally thought to be supported by large-scale interactions among multiple regions and networks within the brain [168]. The strength and organization of these functional networks can be measured by functional connectivity (FC) analysis during tasks and at rest. FC is generally defined by temporal correlations of BOLD activity across different brain regions. Differences in FC have been identified in clinical populations and linked with clinical and behavioral variables [134,169].

In the last decade, FC during a task has been linked to FC during rest [170]. Moreover, it has been shown that inter-individual differences in task-induced blood oxygen level dependent (BOLD) activity can be predicted by resting state properties (positive connectivity strength with task-positive networks and negative connectivity strength with intrinsic resting-state networks such as the default mode network (DMN; [170]). Findings from studies that predict activation during a task from activation at rest suggest that a common mechanism governs neural activity during both rest and task performance. Resting-state activity is typically collected by a five- to twenty-minute fMRI scan while a participant lies still and stares at a black screen with a central fixation cross. Participants are instructed to rest and try not to think of anything in particular. While resting, slow fluctuations in BOLD signal in the absence of a goal-directed action or external input correspond to functionally relevant networks. These networks can provide important information about network functioning during tasks [171]. Here we review both task-based FC and resting state FC in both ASD and DCD.

## 3. Resting State Research 

### 3.1. ASD AON Task and Resting State Connectivity

Altered connectivity in AON systems has been observed in functional task-based studies in ASD and DCD. Many of these studies have reported disrupted connectivity in emotional processing regions in individuals with ASD relative to TD controls. Rudie et al. [172] found that individuals with ASD had significant reductions in connectivity between right IFG and bilateral inferior and superior parietal lobules while passively viewing emotional facial expressions. During an intentional causal attribution task, Kana et al. [173] explored the recruitment of AON areas and found that participants with ASD showed lower activation in the TPJ, right IFG, and left premotor cortex, as well as reduced FC between the TPJ and motor areas. In a combined task-based and resting-state study by Alaerts et al. [13] the authors found that posterior STS (pSTS) hypo-activity during an emotion discrimination task and under-connectivity with fronto-parietal AON at rest were predictive of emotion recognition deficits in adults with ASD. No clinical measures of social severity (e.g., ADOS or ADI-R) were related to any of the resting FC measures, however, the authors did hypothesize that deficiencies in the connections of the visual pSTS may precede alterations downstream in fronto-parietal AON regions [13], which supports the “multiple routes” theories of ASD. In a follow-up study looking at age-related changes in STS FC, the same group reported atypical development of pSTS connectivity with the fusiform, STG and extended insula and angular gyri as well as with other AON regions in ASD [174]. Taken together, these connectivity findings indicate reduced AON connectivity with other social processing regions outside the AON in ASD populations.

Findings from resting-state FC research that focus explicitly within AON regions are more mixed. Fishman, Keown, Lincoln, Pineda, and Müller [175] explored resting-state FC of the AON in children with ASD but found no significant overall group differences compared to a TD cohort. However, within a sample of their most severely symptomatic ASD participants, the authors observed greater connectivity between the right anterior inferior parietal sulcus and left superior frontal gyrus and posterior cingulate cortex in the ASD group compared to the TD group [175]. Shih et al. [176] found differences between TD and ASD children in frontal regions (ASD > TD), but found no significant reduction in FC in a direct comparison between groups in regions associated with imitation. However, structural equation modeling revealed reduced effects of the IPL on the IFG with increased influences on the dorsal prefrontal cortex (dPFC) and on the IFG in ASD participants [176] indicating that children with ASD have atypical connectivity at rest within and between regions of the AON other networks. In a third study by Alaerts et al. [42] the authors explored graph-theoretical properties of the AON using resting-state fMRI data of adolescents and young adults with ASD. The ASD group displayed reduced network density (fewer connections) compared to the TD group, indicating overall lower connectivity of the AON in ASD.

Only one study to date has looked at the relationship between motor skills and resting state connectivity. In a recent study investigating the visual-motor network in ASD, an increase in intrinsic asynchrony was observed between visual and motor systems in children with ASD compared to TD peers [177]. The strength of visual-motor synchrony was related to imitation skills as measured by a modified FAB test in individuals with ASD as well as social skills measured by the Social Responsiveness Scale, Second Edition (SRS-2 [177]). This is the first study to relate social and motor deficits to motor dysfunction in ASD. However, because social and motor skills in ASD may be collinear [10] results have to be interpreted with caution.

Overall, data indicate that AON connectivity appears to be disrupted in ASD both during tasks and while at rest. Both social and motor performance has been associated with FC in ASD suggesting sample variability should be more closely investigated to understand aberrant findings in network function. In general, there is decreased connectivity in the AON system in ASD, however, it should be noted that in other intrinsic resting state networks both hyper- and hypo-connectivity have been observed in ASD (e.g., salience network; [178]) and these findings have been related to social processing [179,180,181].

### 3.2. DCD Task Based and Resting State Connectivity

Very few functional connectivity studies have been published on DCD populations. To the best of our knowledge, no AON task based functional connectivity study has been published. However, one functional connectivity study observed that children with DCD had increased connectivity between frontal and inferior parietal cortices during a Go/No Go task [182]. These findings suggest that functional connections are altered in children with DCD. Similarly, no study has looked at the intrinsic resting state networks, but resting state connectivity at the region of interest (ROI) level has been investigated. The McLeod group [183,184] has published two studies on primary motor cortex connectivity at rest [183,184]. Both studies compared resting state connectivity with the primary motor cortex in children with DCD, ADHD, those with dual DCD+ADHD diagnoses, and TD controls. McLeod et al. [183] found decreased connectivity with structures of the basal ganglia (including the caudate, putamen, and globus pallidus) in children with DCD and/or ADHD, as well as in sensorimotor regions (inferior frontal gyri, and posterior insular cortex) compared to all other groups. The authors suggest that these connectivity disruptions may impact motor attention, planning, and execution processes. The authors also speculate that co-occurrence of neurodevelopmental disorders may have a distinct impact on FC (McLeod et al., [183]).

The same group published a paper on the same data looking at laterality in regions they found to have distributed connectivity with the primary motor cortex in their clinical groups. The authors observed that children with DCD had weaker within-hemisphere connectivity between the primary motor cortex and right putamen compared to the TD and ADHD groups. Interestingly, the DCD group showed stronger connectivity within- and between-hemispheres between the left and right primary motor cortex and sensory-motor cortices [184]. These findings indicate that children with DCD may lack strong within-hemisphere functional connections between the right putamen and the motor cortex, and that this may be specific to DCD (not found in the other clinical groups tested, TD and ADHD). The authors hypothesized that these decreased hemispheric connections may help explain the bimanual coordination deficits observed in children with DCD.

### 3.3. ASD and DCD AON Resting State and Grey Matter Differences

Despite the unbalanced number of studies for the two clinical populations, there is some evidence that supports overlaps in network disruption in ASD and DCD. Specifically, the primary motor cortex connectivity has been identified in both populations as a region indicated in motor deficits. McLeod et al. [184] observed decreased connectivity with the left primary motor cortex and right IFG in the DCD group compared to controls and stronger FC between the left primary motor cortex and the frontopolar cortex in children with co-occurring DCD and ADHD. While primary motor functional organization has been found to be disrupted in ASD [185], no functional primary motor cortex connectivity findings in ASD have been related to motor skills. However, increased cortical thickness in the primary motor cortex has been reported. In a study comparing high-resolution structural scans in ASD with similar TD and clinical populations (ADHD and ASD + ADHD), researchers found that children with ASD only showed increased grey matter volume and surface area in the bilateral primary sensory cortices and in the right primary motor cortex [186]. They also reported that all children with ADHD regardless of ASD diagnosis had increased left inferior parietal cortex surface area. Furthermore, they related these changes to measures of functional motor skills. Impaired praxis (measured by the FAB modified for children) was associated with increased grey matter volume in the right sensory motor cortex in the ASD + ADHD group. Children with comorbid ASD + ADHD had a positive relationship between grey matter volume in the bilateral sensory motor cortices and manual dexterity, whereas children with ASD only showed a negative relationship. The authors suggest that ASD is associated with abnormal morphology of cortical circuits crucial to motor control and learning and that anomalous overgrowth of these regions, particularly the sensory motor cortex, may contribute to impaired motor skill development [186]. Taken together, these studies suggest that motor deficits are related to hyper-connections/overgrowth of the primary motor cortex. Further research is necessary to better understand these results as well as larger intrinsic networks and overlaps between ASD, DCD, and ADHD.

## 4. AON White Matter Organization

Diffusion weighted imaging (DWI) is a technique used to measure the diffusion of water in the brain based on Brownian motion of water. Disruptions in water’s diffusion are thought to reflect microscopic details about the white matter and surrounding tissue architecture. Data from DWI scans provide both qualitative and quantitative information about diffusion that can be used to map and measure the integrity of white matter in the brain. Disruptions in fractional anisotropy (FA; an index of the degree of anisotropy of a diffusion process), mean diffusivity (MD; average diffusion in all directions), and radial diffusivity (RD; index of axonal diameters) can be quantified and associated with functional brain activity [31], as well as behavioral and clinical variables. Even though functional connections can exist between two regions with no direct anatomical connections [187], DWI can provide information regarding organization and microstructure of white matter tracts that link cortical areas to functional circuits. Below we discuss DWI findings of the AON in both ASD and DCD populations.

### 4.1. ASD AON White Matter Organization 

As mentioned above, individuals with ASD may have atypical cortical activation and network function in the AON. It is possible that the white matter tracts that connect grey matter and subcortical regions also may be impaired. The arcuate fasciculus is a bundle of axons that forms part of the superior longitudinal fasciculus (SLF), which is a major white matter association tract and connects the frontal, parietal, and temporal perisylvian cortex [188]. The SLF provides tracts between structures that correspond to the AON [189]. As with functional data, the few studies that focus specifically on AON pathways in ASD report disparate results. When comparing individuals with ASD to TD controls, some studies find DWI differences in the network [31] while others find none [190].

Fishman et al. [31] conducted a large resting state and DWI study on 50 children with ASD and 45 TD children to investigate functional and structural connectivity in brain regions related to imitation in the SLF. The researchers observed reduced FA and increased MD in white matter tracts connecting the bilateral IFG and dorsolateral prefrontal cortex in the ASD group (Fishman et al., [31]). Social severity scores from clinical ASD assessments (ADOS-2 Total score) was negatively correlated with FA of the tract connecting the left IFG and left medial prefrontal cortex, such that lower FA was associated with more severe ASD symptoms. According to a meta-analysis of diffusion tensor imaging (DTI) studies—a DWI technique—in ASD [191], the SLF is one of only a few consistent sites of reduced anisotropy in ASD (with some exceptions, see e.g., Koldewyn et al. [192]). These differences in the microstructural organization of white matter correlated with weaker resting-state FC and greater ASD symptomatology and indicate that white matter tracts in AON may contribute to imitation impairment [31].

Another important tract relevant to ASD is the corpus callosum (CC), a thick tract connecting the brain’s left and right hemispheres. Regions of the CC are topographically organized and many of the subregions connect bilateral areas of the AON. The genu of the CC primarily connects prefrontal association areas [193] as well as anterior inferior parietal regions [194]. The anterior part of the CC connects premotor and supplementary motor cortices, while the mid-body of the CC connects primary motor areas followed by the posterior regions that connect the primary motor and primary sensory areas [195]. Individuals who fail to develop a CC have impairments in language and social cognition that overlap with ASD symptomatology [196]. A diminished CC is another relatively consistent finding in ASD literature [197,198,199,200,201,202]. In a meta-analysis of cross-sectional areas of the CC in ASD, Frazier and Hardan [198] found that the rostral body of the CC connecting premotor and supplementary motor neurons has the greatest reduction. A more recent study, however, did not replicate this finding [203]. Once again, inconsistencies highlight how heterogeneity in ASD needs to be addressed in future research. Correlations have been found between CC measurements and cognitive tests for social deficits [200] however, no studies have looked at both social and motor deficits in relationship to this tract. Nonetheless, these findings collectively indicate significant microstructural abnormalities and alterations in the organization of white matter fibers in ASD, which may translate into functional impairments in brain activation and functional connectivity.

### 4.2. DCD AON White Matter Organization

Some of the earliest neuroimaging papers published on DCD are DWI studies revealing alterations in white matter organization in motor-related networks in children with DCD, particularly in sensorimotor tracts [204,205,206]. Zwicker et al. [206] was the first to identify reduced axial diffusivity in the corticospinal tract and posterior thalamic radiation in children with DCD compared to TD controls. They also found a significant positive correlation between axial diffusivity scores and motor skill level measured by the MABC-2. A second DWI study identified reduced FA in the SLF and the superior/parietal portion of the CC in children with DCD [205]. These findings were unique to DCD compared to children with ADHD, co-occurring DCD and ADHD, or TD peers. Most recently, reduced FA was identified in the internal capsule of children with DCD compared to TD children [204]. Overall, findings indicate that children with DCD have disrupted white matter organization in tracts that connect to motor planning and processing cortical regions. Altered white matter organization was found in young adults with DCD compared to healthy controls using a higher-order CSD-based tractography approach in the bilateral SLF and that the mean apparent fiber density in the SLF was associated motor skills [207]. In a study of adults with a probable DCD diagnosis (pDCD; no formal diagnosis was established) similar results of attenuated FA in the SLF were found in a group of 12 pDCD compared to 11 age-matched controls [32]. These findings suggest that neurobiological alterations along white matter tracts that are known to support motor perception and planning persisted along the lifespan of individuals with DCD or pDCD [32]. Adults with pDCD also had lower FA in the corticospinal tract and lower mean diffusivity in the internal capsule and inferior longitudinal fasciculus [32]. This suggests reduced white matter organization in parietofrontal and corticospinal tracts.

### 4.3. AON White Matter Organization in DCD and ASD 

Broadly, data from DWI studies suggest that the ASD and DCD have a common pattern of reduced FA in SLF. Motor skills have been found to be linked to FA of the SLF in DCD populations [206], and social severity linked to FA in the same region in ASD samples. No studies have yet explored how social skills relate to the SLF in DCD or motor skills in ASD nor compared the two groups directly. It is possible that reduced SLF integrity has discrete patterns for each group.

To our knowledge, only one neuroimaging study has compared ASD (high functioning) and DCD. Instead of looking at white matter microstructure, Caeyenberghs et al. [208] used graph theory analysis to relate cortical structures to motor performance in children with DCD (*n* = 11), ASD (*n* = 15), co-occurring ASD and DCD (ASDd, *n* = 8), and TD (*n* = 19). Despite the relatively small sample size, behavioral and structural network parameters were detected. Behaviorally, children with any DCD diagnosis (DCD, ASDd) were found to have poorer performance on motor ability (indexed by the MABC-2 [162]), and visual motor integration (indexed by the Beery VMI [209]) compared to ASD and TD participants. At the neurological level, the ASD group was found to have the most abnormal network connectivity; notably, increased normalized path length and higher values of clustering coefficient, while children with DCD displayed a global network organization that was similar to TD children. These findings are inconsistent with Rudie et al. [210] who reported decreased long-range FC in ASD. Caeyenberghs and colleagues [208] concluded that increased clustering and path lengths found in ASD reflect unbalanced and inefficient network organization. At the nodal level, the ASD group displayed increased clustering coefficient in the right IFG orbitalis and decreased clustering coefficient in the right cingulate gyrus compared to the TD cohort. Unique to the DCD group was increased clustering coefficient in the lateral orbitofrontal cortex—a part of the expanded limbic system. Children with ASDd had more widespread deviations from typical patterns of cortical thickness than those seen in children with only DCD or only ASD, such as alterations of clustering coefficients in the pars orbitalis of the right IFG, paralimbic regions, primary motor areas, and association areas compared to the TD group. Notably, the ASDd group showed increased clustering of the AON regions (left IFGpo) compared to the ASD group as a whole. Together, these results indicate that abnormal AON function in ASD may be underscored by an additive effect of both social and motor deficits since the IFGpo had typical clustering coefficient that was not altered in the ASD only or DCD only groups. Together, diffusion and cortical thickness findings suggest that individuals with ASD have more severe structural abnormalities and that individuals with ASDd symptomatology may exhibit unique deficits compared to their peers with either ASD or DCD.

## 5. Conclusions and Future Directions

This review covered current literature and theories of AON across three levels of neurobiology in both ASD and DCD populations: task, resting state networks, and white matter organization. With limited published research on the DCD population, it is hard to point to any definitive findings comparing the two clinical populations. What is already apparent, however, is the complexity of the AON and motor systems networks. The AON is connected to several networks that may modulate its function (e.g., sensorimotor, fronto-parietal, cerebellar, and reward networks). Further, the various imitation and motor impairments that may manifest in children with ASD indicates that no common neurobiological dysfunction may be associated with ASD motor impairments in general. Findings from dual diagnoses (e.g., ASD + ADHD, DCD + ADHD) research suggests that individual symptomatological differences can have large implications for understanding neural functioning and behavior in ASD. It is clear from ASD fMRI task, resting state, and diffusion research that social deficits are linked to AON functioning during fMRI observation and imitation tasks. What is less clear is how motor and imitation skills correspond to AON function in ASD, however, a few findings from resting state studies that have investigated this association suggest that there is a significant relationship. Specifically, one study directly investigating imitation skills in ASD related imitation deficits to weaker connectivity in motor areas [177]. DWI work further supports this association between motor skills and structural connectivity in a few DCD diffusion papers [205,206]. Because DCD is a common comorbid condition in ASD, understanding how motor skills relate to social skills will help identify behavioral and neurological patterns discretely associated with ASD. As mentioned above, to date only one neuroimaging study has compared ASD and DCD populations [208]. Future research should examine motor and social networks across these two populations as well as with a typical control group.

## 6. Limitations

There are several limitations to this review. First, the volume of ASD publications related to the AON vastly outnumbers the DCD publications. This imbalance is compounded by the average difference in sample size with the average study’s sample size for ASD being much larger than in DCD research. Studies with larger DCD sample sizes are needed to make appropriate comparisons. Secondly, this review is restricted to AON-related studies (with a few exceptions) and does not cover other all structural and volumetric literature in ASD and DCD. Further examination of these two populations across other networks such as mentalizing and emotional-related networks is vital to understanding the extent to which motor impairment may affect social impairment in both disorders. Additionally, this paper does not discuss other co-morbid symptoms and diagnosis that may impact motor skills in ASD and DCD such as cerebral palsy and intellectual impairments. Future work should also explore ASD-DCD differences in other motor-related regions such as the cerebellum and subcortical regions such as the basal ganglia and putamen. Previous work has posited a pathological role for the cerebellum in ASD [133,211] as well as in DCD [212] which makes it an exceptionally relevant region to study.

## Figures and Tables

**Table 1:** Task-based functional magnetic resonance imaging studies investigating developmental coordination disorder (DCD).

Author	Population	Sample Size	Age (years)	DCD Inclusion	Motor measures	Task	AON Results (DCD vs. TD)
Querne et al. 2008	DCD, TD	*n* = 19 DCD = 9 TD = 10	8–13	DSM-IV criteria for DCD	Sensori-motor functions scales (NEPSY)	Go/no-go	none
Kashiwagi et al. 2009	DCD, TD	*n* = 24 DCD = 12 TD = 12	9–12	Below 15th percentile > 2 positive SNS Parent interview	MABC	Visuomotor task (visually guided tracking task)	Decreased activation: left posterior parietal cortex and left postcentral gyrus
Zwicker et al. 2010	DCD, TD	*n* = 14 DCD = 7 TD = 7	8–12	Below 15th percentile	MABC-2	Trail-tracing task	Decreased activation: IFG Increased activation: left IPL
Zwicker et al. 2011	DCD, TD	*n* = 14 DCD = 7 TD = 7	8–12	Below 15th percentile	MABC-2; DCDQ	Trail-tracing task	Decreased activation: bilateral IPL
Debrabant et al 2013	DCD, TD	*n* = 34 DCD = 17 TD = 17	7–10	Below 5th percentile	MABC-2	Visuomotor reaction time task	none
Licari et al. 2015	DCD, TD	*n* = 26 DCD = 13 TD = 13	8–10	Below 5th percentile	MABC-2	*Finger sequencing task Hand clenching task	Decreased activation: left IFG
Reynolds et al. 2015	DCD, TD	*n* = 26 DCD = 14 TD = 12	8–12	Below 16th percentile	MABC-2	*Imitative finger-sequencing task (1) observation, (2) action execution, and (3) action imitation	None Across decreased IFG during imitation compared to other tasks.
Reynolds et al. 2017	DCD, TD	*n* = 19 DCD = 10 TD = 9	8–12	Below 16th percentile	The Postural Praxis and Sequencing Praxis (SIPT), MABC-2, hand rotation task	*Finger abduction/adduction (1) action observation; (2) motor imagery; (3) action execution; and (4) action imitation.	none
Biotteau et al. 2017	DD, DCD, DD+DCD	*n* = 48 DCD = 16 DD =16 DD + DCD = 16	8–12	Below 5th percentile, and outside diagnosis	MABC-2	Procedural learning finger-tapping task	DCD vs. DD: Increased activation: bilateral premotor cortex
Kashuk et al. 2017	pDCD, TD	*n* = 23 pDCD = 12 TD = 11	18-40	Below 15th percentile on total, fine motor, or gross motor scores and functional deficit on ADC	MAND ADC	Hand rotation task (no execution/imitation)	none
Thornton et al., 2018	DCD, TD, ADHD, DCD+ADHD	*n* = 48 DCD = 9 TD = 20 ADHD = 20 DCD + ADHD = 18	8–17	At or below 16th percentile and functional deficit on DCDQ	MABC-2, DCDQ	Go/no go task	N/A

AON = action observation network, DD = Developmental Dyslexia, DCD = Developmental Coordination Disorder, pDCD= probable DCD, MABC = Movement Assessment Battery for Children, MAND = McCarron Assessment of Neuromuscular Development, DCDQ = Developmental Coordination Disorder Questionnaire, IFG = Inferior frontal gyrus, IPL = Inferior parietal lobules. * Action Observation or imitation task.
